# New Mechanistic Insights Into Nobiletin’s Protective Effects Against LPS‐Induced Hyperinflammatory Acute Lung Injury in Rats: Targeting miR‐200, AT1R/p38 MAPK, and HMGB1/NF‐κB mTOR Pathways

**DOI:** 10.1155/omcl/3253224

**Published:** 2026-10-21

**Authors:** Rehab Ahmed Ahmed El-Shaer, Sarah Ibrahim, Norhan Ahmed AbuoHashish, Amira K. Eltokhy, Heba Bassiony Ghanem, Rowida Raafat Ibrahim, Passant Medhat Hewady, Omnia Safwat El-Deeb, Eman E. Farghal, Radwa Eissa, Marwa M. Shalaby, Marwa Mahmoud Awad

**Affiliations:** ^1^ Physiology Department Faculty of Medicine Tanta University Tanta Egypt tanta.edu.eg; ^2^ Basic Science Department Al-Rayan National College of Medicine Al Madinah Al Munawwarah Saudi Arabia; ^3^ Human Anatomy and Embryology Department Faculty of Medicine Tanta University Tanta Egypt tanta.edu.eg; ^4^ Pharmacology Department Faculty of Medicine Tanta University Tanta Egypt tanta.edu.eg; ^5^ Medical Biochemistry Department Faculty of Medicine Tanta University Tanta Egypt tanta.edu.eg; ^6^ Clinical Laboratory Sciences Department College of Applied Medical Sciences Jouf University Sakaka Saudi Arabia ju.edu.sa; ^7^ Clinical Pathology Department Faculty of Medicine Tanta University Tanta Egypt tanta.edu.eg; ^8^ Microbiology and Immunology Department Faculty of Medicine Tanta University Tanta Egypt tanta.edu.eg

**Keywords:** ACE2/Ang1–7, ALI, hyperinflammation, miR-200, NF-κB, nobiletin

## Abstract

**Background:**

Acute lung injury (ALI) and acute respiratory distress syndrome (ARDS) are severe inflammatory lung disorders associated with high morbidity and mortality. Dysregulation of the renin–angiotensin system (RAS) and proinflammatory pathways play an indispensable role in ALI progression.

**Aim:**

This study aimed to investigate the protective potential of nobiletin (NOB) in mitigating ALI and hyperinflammatory lung injury.

**Material and Methods:**

The study enrolled 32 male albino rats allocated into four groups: (Control, NOB, lipopolysaccharide [LPS], and NOB + LPS groups). Rats received NOB 20 mg/kg by oral gavage for 1 week; on the seventh day, a single dose of 15 mg/kg LPS was given by intranasal instillation. After 24 h of LPS administration, plasma and lung tissue samples were analyzed for inflammatory cytokines, mitochondrial complex‐I, oxidative stress markers, and molecular signaling pathways.

**Results:**

NOB administration significantly downregulated miR‐200 expression, associated with ACE2 activation and attenuated AT1R‐mediated inflammatory responses. It also decreased NF‐κB and p38 MAPK signaling, reducing pulmonary and systemic levels of tumor necrosis factor‐alpha (TNF‐α), interleukin‐6 (IL‐6), granulocyte monocyte‐colony stimulating factor (GM‐CSF), and interleukin‐17A (IL‐17A). Additionally, NOB improved the high mobility group box 1 (HMGB1)/NF‐κB/mammalian target of rapamycin (mTOR) pathway, and increased mitochondrial complex‐I may be consistent with improved tissue preservation.

**Conclusion:**

NOB exerts protective effects against ALI by improving inflammatory cytokine, oxidative stress, miRNA‐200, mitochondrial complex‐I, and ACE2, highlighting its potential as a protective candidate for ALI and hyperinflammatory‐associated lung injury.

## 1. Introduction

Acute lung injury (ALI) is a severe inflammatory pulmonary disorder characterized by diffuse alveolar damage, increased vascular permeability, pulmonary edema, and impaired gas exchange. Excessive production of proinflammatory cytokines, including tumor necrosis factor‐alpha (TNF‐α) and interleukin‐6 (IL‐6), plays a central role in disease progression by amplifying tissue injury and compromising pulmonary function [1].

The COVID‐19 pandemic highlighted the clinical significance of hyperinflammatory lung injury and emphasized the need to better understand the molecular mechanisms underlying ALI. Although SARS‐CoV‐2 infection possesses unique virological and immunological characteristics, dysregulated inflammatory responses and excessive cytokine production are common pathological features shared with several forms of ALI. Therefore, insights gained from experimental ALI models may contribute to a better understanding of inflammatory lung diseases while recognizing the inherent limitations of each experimental model [2, 3].

To develop a deeper insight into the fundamental mechanisms, Al‐Ani et al. [1] have developed an animal model that mimics the hyperinflammatory pathophysiology observed in these patients.

Lipopolysaccharide (LPS) is one of the most widely used experimental agents for inducing ALI in rodents, enabling the investigation of inflammatory mechanisms and the evaluation of potential therapeutic interventions. LPS activates Toll‐like receptor‐4 (TLR4) signaling, leading to inflammatory cell recruitment, disruption of the alveolar‐capillary barrier, increased vascular permeability, pulmonary edema, and excessive production of proinflammatory cytokines [4]. Although this model reproduces many inflammatory and histopathological features of ALI, it does not fully recapitulate the virological and immunological complexity of viral infections, such as SARS‐CoV‐2.

Dysregulation of the renin–angiotensin system (RAS) is another important contributor to ALI pathogenesis through its effects on inflammation, oxidative stress, vascular permeability, and pulmonary injury. Emerging evidence has demonstrated that microRNAs, particularly miR‐200, regulate ACE2 expression and thereby influence the balance between the deleterious ACE/Ang II/AT1R axis and the protective ACE2/Ang‐(1–7)/Mas receptor axis [5].

Activation of the AT1R/p38 MAPK signaling pathway plays a pivotal role in amplifying inflammatory responses during ALI by promoting the production of proinflammatory mediators such as TNF‐α and IL‐6. Persistent activation of this pathway contributes to endothelial dysfunction, pulmonary edema, and progressive lung injury [6].

In addition, these cytokine dysregulations are linked to damage‐associated molecular patterns (DAMPs), particularly high mobility group box 1 (HMGB1). Elevated HMGB1 levels in ALI patients correlate with heightened inflammation. HMGB1, released from infected cells, binds to pattern recognition receptors like TLR4, activating nuclear factor kappa‐light‐chain‐enhancer of activated B cells (NF‐κB) signaling and inducing proinflammatory cytokines [7].

Meng et al. [8] highlighted the role of HMGB1‐TLR4/NF‐κB and PI3K/Akt/mammalian target of rapamycin (mTOR) pathways as inflammatory signal pathways in the pathogenesis of ALI.

A polyethoxylated flavone nobiletin (NOB) derived from citrus peels exhibits diverse pharmacological properties, including anticancer, anti‐inflammatory, antioxidant, and anti‐insulin resistance effects [9]. NOB has been demonstrated to effectively decrease cell proliferation and viability in breast cancer cells while also exhibiting nephroprotective properties by attenuating oxidative stress in experimental models of acute kidney injury [10].

NOB has demonstrated diabetic attenuation effects by restoring blood glucose and insulin to normal levels [11], improving kidney function, and reducing injury in diabetic rat models. It has also shown potential in reducing metabolic syndrome complications induced by a high‐fat diet [12].

In addition, Iampanichakul et al. [13] reported that NOB improved the left ventricular function and mitigated remodeling in hypertensive rats by reducing oxidative stress and inhibiting the Ang II/AT1R/JAK/STAT pathway. Its RAS‐inhibitor and antioxidant properties also attenuate renal injury associated with renovascular hypertension through amelioration of the angiotensin II receptor type 1 (AT1R/Nox4) pathway.

Despite the growing evidence supporting the anti‐inflammatory and antioxidant properties of NOB, its effects on the integrated miR‐200/ACE2/AT1R/p38 MAPK/HMGB1/NF‐κB/mTOR signaling network in LPS‐induced ALI remain poorly understood. Therefore, we hypothesized that NOB could have a protective effect against LPS‐induced ALI through coordinated modulation of these interconnected inflammatory and survival pathways. Accordingly, the present study aimed to investigate the protective effects of NOB against LPS‐induced ALI and to explore the molecular mechanisms underlying its actions.

## 2. Materials and Methods

### 2.1. Chemicals and Drugs

NOB powder (purity ≥ 97%, N1538‐10 MG) and LPS (lyophilized powder, L2630‐25 MG) were acquired from Sigma‐Aldrich Co. (St. Louis, MO, USA). High‐grade analytical chemicals and solvents were used in all analyses.

### 2.2. Animals

Thirty‐two male albino rats, each aged between 10 and 12 weeks and weighing ~190–210 g, were selected for the experiment. They were sourced from the animal care facility at the Faculty of Medicine, Tanta University, Egypt. To ensure their well‐being, the rats were housed in clean, well‐ventilated wire cages, with four animals per cage, under controlled environmental conditions, including a 12 h light/dark cycle. They were given 1 week to acclimate to their surroundings before the experiment began. Throughout the study, they had unrestricted access to standard food and fresh water. All experimental procedures were approved by the Medical Research Ethics Committee at the Faculty of Medicine, Tanta University (Approval Code 36264PR446/11/23), following the ethical guidelines set by the National Institutes of Health for the care and use of laboratory animals (NIH Publication Number 8023, revised 1996).

### 2.3. Experimental Design

After being acclimatized, rats were randomly divided equally into four groups, each containing eight rats.

Control group: rats received 1 mL of normal saline by oral gavage for 1 week, and on the seventh day, rats were intranasally injected with 200 µL of phosphate‐buffered saline solution (PBS) without LPS.

NOB group: rats received 20 mg/kg NOB by oral gavage for 1 week [14], and on the seventh day, rats were intranasally injected with 200 µL of PBS without LPS.

LPS group: rats were given 1 mL of saline by oral gavage for a week. On the seventh day, ALI was triggered by delivering a single 15 mg/kg dose of LPS via intranasal instillation [1] in a 200 µL sterile PBS solution. The LPS mixture was carefully administered through the rats’ nasal passages using a pipette. To ensure an even distribution across lung tissues, the animals were gently shaken horizontally for about half a minute [8].

NOB + LPS group: rats received 20 mg/kg NOB by oral gavage for 1 week [14]. and on the seventh day, a single dose of 15 mg/kg LPS was administered by intranasal administration as mentioned before [8].

### 2.4. Blood and Tissue Sampling

The mortality rates among all groups were recorded daily for 10 days. Separate cohorts of animals were utilized for the 10‐day survival analysis and the day 3 biochemical/histological assessments to prevent confounding tissue data with mortality‐related artifacts.

Cohort 1 (survival): 8 animals per group. They were monitored for 10 days solely to see who lives and who dies. No tissues were taken from them on day 3.

Cohort 2 (tissues): 8 separate animals per group. They were all humanely euthanized on day 3 specifically to harvest their organs for histopathology and biochemical tests.

After 3 days of LPS administration, rats were anesthetized with sodium barbiturate (60 mg/kg, i.p.) [15]. With their hearts still beating, blood was collected via cardiac puncture, and blood samples were collected in tubes containing 3.2% sodium citrate, ensuring a precise ratio of anticoagulant to blood (1:9, respectively). The samples were thoroughly mixed before undergoing centrifugation at 4000 rpm for 10 min. Immediately after separation, plasma was carefully extracted and stored under different conditions: at room temperature (22 ± 3°C), refrigerated at 4°C, and frozen at −20°C to maintain the sample integrity.

Once the necessary blood samples were secured, the rats were humanely euthanized using cervical dislocation. Lung tissues were collected and dissected for further investigation. Three portions of lung tissue were prepared; one portion was preserved in 10% formalin for histopathological and immunostaining assessments, while another was homogenized in PBS (50 mM, pH 7.4) for ELISA preparation. To ensure long‐term viability for future molecular analysis, another two portions were stored at −80°C, designated specifically for biochemical analysis, mitochondrial, nuclear, and RNA extraction.

### 2.5. Lung Wet‐to‐Dry Weight Ratio

After sacrifice, the lungs were carefully removed, ensuring that the lobes remained intact without lavage. Each lung was weighed immediately to determine its wet weight. To extract the dry weight, the lung tissue was then placed in an oven at 80°C for 48 h, allowing all moisture to evaporate. The final wet‐to‐dry weight ratio was calculated to evaluate the extent of tissue edema, providing insight into fluid accumulation in the lungs [14].

### 2.6. Estimation of Prothrombin Time (PT), Activated Partial Thromboplastin Time (APTT), Plasma Plasminogen Activator Inhibitor (PAI‐1), D‐Dimer, and Ferritin Levels

PT and APTT were measured following established protocols reported by Goyal et al. [16]. To assess coagulation and inflammatory responses, plasma levels of PAI‐1 and D‐dimer were analyzed using specialized Rat ELISA kits from MyBioSource, Inc., San Diego, United States. Catalog # MBS2023901 and MBS725327, respectively. Additionally, ferritin concentration was determined using the Rat Ferritin ELISA Kit from Thermo Fisher Scientific (Waltham, USA; Catalog # EEL129), adhering strictly to the manufacturer’s guidelines for accuracy and consistency.

### 2.7. Assessment of TNF‐α, IL‐6, Granulocyte Monocyte‐Colony Stimulating Factor (GM‐CSF) and Interleukin‐17A (IL‐17A) in Plasma and Lung Tissue

The levels of TNF‐α, IL‐6, GM‐CSF, and IL‐17A in plasma and lung tissue homogenate samples were evaluated by Rat ELISA kits (MyBioSource, Inc., San Diego, United States. Catalog #MBS2507393, MBS269892, MBS2507633, and MBS704126), respectively. All procedures were performed according to the manufacturer’s instructions.

### 2.8. Oxidative Stress Markers: Malondialdehyde (MDA) Levels, Superoxide Dismutase (SOD), and Myeloperoxidase (MPO) Activities Assay in Lung Tissue

To measure MPO activity and MDA levels, which reflect lipid peroxidation in ALI, lung tissue homogenates were analyzed using Rat ELISA kits from MyBioSource, Inc., San Diego, USA. The specific catalog numbers used were MBS2709401 for MPO and MBS268427 for MDA. SOD activity was assessed by a commercial kit (Biodiagnostic, Worcestershire, UK) Catalog Number (SD 25 20). Lung samples were carefully homogenized in ice‐cold saline to maintain their stability. The mixture was then spun at high speed (12,000 g) at 4°C for 20 min to separate the components effectively. Afterward, the clear supernatant was collected and promptly stored at a deep freeze temperature of −80°C to preserve its integrity for further analysis.

### 2.9. Determination of Surfactant Protein (SP‐A), Angiotensin II and Angiotensin 1–7 Levels and ACE2 Activity in Lung Tissue

The pulmonary contents of SP‐A, angiotensin 1–7, and angiotensin II contents were investigated using Rat ELISA kits (MyBioSource, Inc. San Diego, United States; Catalog # MBS703468, MBS705139, and MBS2022279, respectively) according to the manufacturer’s instructions, while the ACE2 activity assay kit was used for investigation of ACE2 activity in lung tissue according to the manufacturer’s instructions (Bioassay Technology Laboratory, Shanghai Korain Biotech, Shanghai, China, Catalog # E0968Ra).

### 2.10. Determination of Inflammatory and Mitochondrial Markers

#### 2.10.1. Quantitative Real‐Time Polymerase Chain Reaction (qRT‐PCR) for miR‐200, AT1R, and HMGB‐1 Expression Essays

RNA extraction and gene expression analysis were performed on lung tissue samples collected from each group. Using the Gene JET RNA Purification Kit (Thermo Scientific, USA; Catalog Number K0731), high‐quality total RNA was carefully isolated. The extracted RNA was then assessed for concentration and purity by measuring its optical density (OD) at 260 nm, with the OD260/280 ratio checked using an Implen NanoDrop Spectrophotometer (Implen, USA) to ensure integrity.

Following isolation, 5 μg of RNA was converted into complementary DNA (cDNA) using the RevertAid H Minus Reverse Transcriptase Kit (Thermo Scientific, USA; Catalog Number EP0451). The resulting cDNA was amplified using TOPreal qPCR 2x PreMIX (SYBR Green/ROX qPCR Master Mix) (Thermo Scientific, USA; Catalog Number K0221). Amplification and quantification of miR‐200 gene expression were performed using the Rotor‐Gene Q5 plex System (Qiagen, Germany).

For further analysis, the generated cDNA was subjected to qRT‐PCR to assess the expression of multiple genes, including miR‐200, AT1R, and HMGB‐1. This was conducted using an Applied Biosystems Step One Plus real‐time PCR system (Waltham, USA). Specific primer sequences corresponding to each gene are detailed in Table 1.

**Table 1:** Forward and reverse primers sequence for the quantitative qRT‐PCR for the candidate genes.

Gene	Forward primer (5′–3′)	Reverse primer (5′–3′)
miR‐200	GCGGCGGTGGCAGTGTCTTAGC	ATCCAGTGCAGGGTCCGAGG
snRNA U6	AGCAGATCTTGAGGTTACGGA	GGCATGAGGCAAACAGTCT
AT1R	TAACAACTGCCTGAACCCTC	GCGTGCTCATTTTCGTAGAC
HMGB‐1	AGTTTCCTGAGCAATCCGTAT	TGTATCCCCAAAAGCGTGAG
GAPDH	CAACTCCCTCAAGATTGTCAGCAA	GGCATGGACTGTGGTCATGA

The thermal cycling conditions were optimized as per the manufacturer’s instructions, starting with an initial DNA polymerase activation at 95°C for 15 min, followed by repeated three‐step cycling for 45 cycles, including denaturation, annealing, and extension steps at specific temperatures. A melting curve analysis was also performed to verify the specificity and identity of the PCR products. To ensure accuracy and eliminate bias, negative controls were included in every PCR run. Each sample was processed blindly and analyzed in duplicate.

Relative gene expression levels were calculated using the 2^−ΔΔCt^ method [17], with normalization against snRNA U6 and GAPDH serving as internal reference controls for miRNA and mRNA quantification, respectively.

#### 2.10.2. Determination of Lung Tissue p38 MAPK, and mTOR Protein Level

To measure mitogen‐activated protein kinase (p38 MAPK) and mTOR protein levels in lung tissue, the homogenates were analyzed using Rat ELISA kits from MyBioSource, Inc., San Diego, USA. Catalog # MBS267271 and MBS8808126, respectively.

#### 2.10.3. Mitochondrial Samples Analysis and Complex I Activity

Mitochondrial fractions were isolated from lung tissue homogenates via differential centrifugation at 4°C. Soft tissue samples were gently homogenized in an ice‐cold mitochondrial isolation buffer consisting of 250 mM sucrose, 10 mM Tris‐HCl, and 1 mM EDTA (pH 7.4), supplemented with a 1× protease inhibitor cocktail. The homogenate was first centrifuged at 2000 × g for 10 min at 4°C to sediment unbroken cells and nuclear debris. The resulting supernatant was collected and subjected to a secondary centrifugation step at 5000 × g for 20 min at 4°C to pellet the crude mitochondrial fraction. The supernatant was discarded, and the mitochondrial pellet was resuspended in isolation buffer [18].

Mitochondrial complex I enzyme activity was subsequently quantified using the Complex I Enzyme Activity Microplate Assay Kit (Colorimetric, Abcam, Cat# ab109721) according to the manufacturer’s instructions. Briefly, isolated fractions were detergent‐extracted, and equal amounts of protein were loaded into the microplate wells precoated with highly specific anti‐Complex I monoclonal antibodies to immobilize the enzyme. Complex I activity was assesed by photometric assay at room temperature by monitoring the oxidation of NADH to NAD^+^ coupled to the simultaneous reduction of a reporter dye, measured as an increase in absorbance at OD 450 nm over 30 min [19].

### 2.11. Histological Staining and Assessment of Lung Pathology

Lung tissues were carefully preserved in 10% formalin for an entire day, ensuring optimal fixation. The samples then underwent a stepwise dehydration process using graded alcohol concentrations before being cleared and embedded in paraffin, adhering to well‐established histological protocols. Thin sections of 4 μm thickness were prepared from the paraffin blocks and stained with hematoxylin and eosin (H&E) to provide a clear visualization of lung architecture and structural modifications [20].

To further investigate the presence of alveolar hemorrhage, Perl’s stain was applied to highlight hemosiderin‐laden macrophages, a key indicator of bleeding in lung tissue [21]. The staining process involved immersing the sections in a 20% diluted hydrochloric acid solution, which liberated ferric iron, followed by exposure to a 10% aqueous potassium ferrocyanide solution. The sections were counterstained with eosin, yielding deep‐blue deposits where ferric iron accumulated, while the nuclei took on a reddish hue for contrast. The stained lung sections were captured and analyzed using a Leica Qwin 500 C image analyzer, ensuring a detailed examination across 10 distinct, nonoverlapping fields per group [22].

Histopathological evaluation focused on key microscopic findings, including leukocytic infiltration, thickened interalveolar septa, alveolar hemorrhage (evidenced by the presence of hemosiderin‐laden macrophages in Perl’s blue‐stained sections), and hyaline membrane formation. The severity of lung injury was graded on a 5‐point scale, ranging from absent to severe (0, none; 1, minimal; 2, mild; 3, moderate; 4, marked; and 5, severe), while the distribution of these pathological changes was also categorized on a five‐tiered scale, spanning from focal to diffuse involvement (0, none; 1, focal; 2, locally extensive; 3, multifocal; 4, multifocal with coalescence; and 5, diffuse). For accuracy and consistency, an equivalent number of lung sections were assessed per subject, and the overall lung injury score was derived by combining severity and distribution scores for each rat within a group. Finally, the average total score was calculated to compare the extent of lung injury across experimental groups.

### 2.12. Immunohistochemical Staining of NF‐κB.

For immunohistochemistry, 5 µm‐thick deparaffinized tissue slices were prepared with precision. To retrieve antigens, the sections were carefully boiled for 10 min in a 10 mM citrate buffer solution at pH 6, allowing optimal exposure of target proteins. After this heat‐induced process, the slides were left to cool at room temperature for 20 min, ensuring gradual stabilization before the next step.

The sections were incubated with a rabbit polyclonal antibody against NF‐κB (MyBioSource, Catalog Number MBS9701121). This transcription factor plays a central role in driving cytokine production and is heavily involved in the inflammatory cascade. After primary antibody incubation, the sections underwent counterstaining with Meyer’s hematoxylin, adding contrast for better visualization.

To quantify the immunohistochemical expression, the stained slides were analyzed using ImageJ software (version 1.46a, NIH, Bethesda, MD, USA). The assessment focused on the mean surface area percentage of NF‐κB immunostaining. This parameter was examined across 10 distinct, nonoverlapping fields (200× magnification), providing a comprehensive evaluation of NF‐κB expression in lung tissue samples.

### 2.13. Transmission Electron Microscopy (TEM)

Tiny lung tissue fragments, roughly 1 mm^3^, were carefully excised and immediately submerged in 2.5% glutaraldehyde for fixation, ensuring structural preservation. After about 6 h, the samples were thoroughly rinsed with 0.1 M PBS (pH 7.4) to remove excess fixative. To enhance stability, a postfixation step was carried out using 1% osmium tetroxide, buffered to pH 7.4 with PBS, and maintained at 4°C for 1–2 h. Another PBS rinse followed to clear any remaining chemicals. The dehydration process involved gradually increasing ethanol concentrations, ensuring meticulous water removal before transitioning to propylene oxide for final clearing. Once prepped, the specimens were embedded in Araldite 502, forming gelatinous capsules that underwent polymerization at 60°C, solidifying their structure.

For microscopic evaluation, semi‐thin sections (~1 mm thick) were stained with toluidine blue, aiding in orientation. For deeper structural insight, ultra‐thin sections (~100 nm thick) were carefully sliced using an ultramicrotome and mounted onto uncoated copper grids. The final step involved double staining with uranyl acetate and lead citrate, heightening the contrast for electron microscopy. Each sample was visualized, and three to five random micrographs were captured per section using a JEM‐1011‐JEOL transmission electron microscope operating at 80 kV [23].

### 2.14. Statistical Analysis

The normality of all datasets was assessed using the Shapiro–Wilk test. Normally distributed data are presented as mean ± SD and were analyzed using one‐way ANOVA, followed by Tukey’s post hoc test. Nonnormally distributed data are presented as boxplots showing the median and interquartile range (25th–75th percentiles) and were analyzed using the Kruskal–Wallis test, followed by Dunn’s pairwise post hoc test.

To ensure precise statistical processing, SPSS software (version 20.0 for Windows, Chicago, IL, USA) was utilized for all calculations. A two‐tailed *p*‐value < 0.05 was considered statistically significant. For highly significant comparisons, values were presented as *p* < 0.001 or *p* < 0.0001 according to the statistical output.

## 3. Results

### 3.1. Mortality Rate and Survival Analysis Among All Groups Studied

Mortality and survival analyses were assessed among the studied groups. There was no mortality among the Control and NOB groups, while the mortality rates were 37.5% in the LPS group, and 12.5% in the NOB + LPS group, as demonstrated in Table 2 and Figure 1.

**Figure 1:**
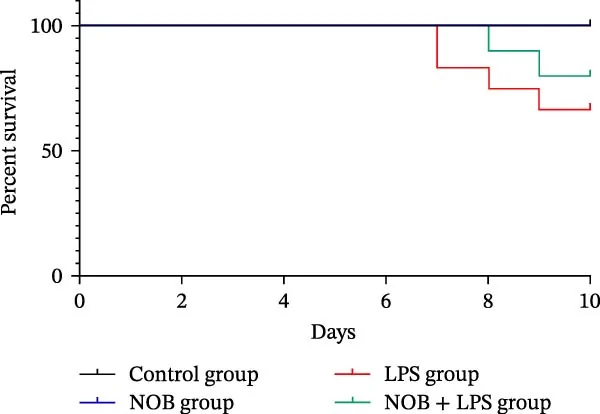
Kaplan–Meier survival analysis with group comparisons using the log‐rank test. LPS, lipopolysaccharide; NOB, nobiletin.

**Table 2:** Mortality rate among all studied groups.

Day	Group			
	Control group	NOB group	LPS group	NOB + LPS group
Day 1	8	8	8	8
Day 10	8	8	5	7
Mortality rate	0%	0%	37.5%	12.5%

*Note:* The number of rats enrolled in each group at day 1 (the start) and day 10 (the end) of the experiment.

### 3.2. Effect of NOB in Combating LPS‐Induced Pulmonary Edema

Lung insult triggered by a single intranasal injection of LPS (15 mg/kg) resulted in a substantial increase in capillary leakage, resulting in the development of pulmonary edema. Pulmonary edema was assessed in the present study by the lung wet/dry ratio. It is noted in Figure 2 that the lung wet/dry ratio in the LPS group was significantly higher than that those in the Control and NOB groups. Conversely, pretreatment with NOB (20 mg/kg) significantly reduced the wet/dry ratio in the NOB + LPS group when compared with the LPS group.

**Figure 2:**
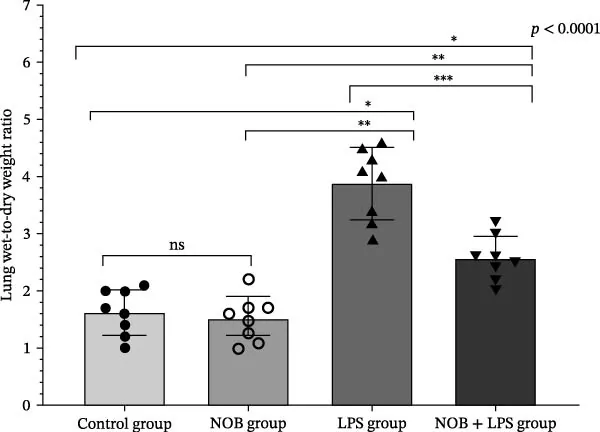
Impact of NOB on lung W/D ratio in LPS‐induced ALI mice. The data are illustrated as mean ± SD, providing a clear representation of variability. Statistical analysis was carried out using one‐way ANOVA, followed by Tukey’s post hoc test for multiple comparisons. ns denotes nonsignificant,  ^∗^ indicates a significant difference compared to the Control group, while  ^∗∗^ denotes significant differences compared to the NOB group, and  ^∗∗∗^ denotes a significant difference compared to the LPS group. LPS, lipopolysaccharide; NOB, nobiletin.

### 3.3. Effect of NOB on PT, APTT, Plasma Levels of PAI‐1, D‐Dimer, and Ferritin Levels During LPS‐Induced ALI

Table 3 shows that, 3 days following a single intranasal injection of LPS (15 mg/kg), there was significant prolongation in PT and APTT with a concomitant significant increase in plasma levels of PAI‐1, D‐Dimer, and ferritin when compared with the Control and NOB groups. In contrast to the LPS group, PT and APTT were significantly shortened with a concomitant significant decrease in plasma levels of PAI‐1, D‐Dimer, and ferritin by pretreatment with NOB at a dose of 20 mg/kg for 1 week prior to LPS administration.

**Table 3:** Effects of NOB on prothrombin time (PT), activated partial thromboplastin time (APTT), plasma levels of plasminogen activator inhibitor (PAI‐1), D‐Dimer, and ferritin levels.

Parameters/groups	Control group (*n* = 8)	NOB group (*n* = 8)	LPS group (*n* = 8)	NOB + LPS group (*n* = 8)	*F* ratio	*p*‐Value
PT (s)	19.25 ± 2.82	18.76 ± 3.66	54.88 ± 3.98^ab^	29.25 ± 3.85^abc^	176.54	*p* < 0.0001
APTT (s)	67.63 ± 6.89	64.13 ± 6.08	174.00 ± 6.00^ab^	127.75 ± 4.46^abc^	630.98	*p* < 0.0001
PAI‐1 (ng/mL)	6.28 ± 1.17	6.70 ± 0.919	17.30 ± 1.46^ab^	11.23 ± 0.93^abc^	162.215	*p* < 0.0001
D‐Dimer (ng/mL)	2.18 ± 0.45	1.80 ± 0.33	14.88 ± 2.03^ab^	8.00 ± 1.85^abc^	153.27	*p* < 0.0001
Ferritin (ng/mL)	67.38 ± 5.04	64.63 ± 4.37	161.88 ± 5.64^ab^	102.13 ± 4.12^abc^	702.09	*p* < 0.0001

*Note:* The data are illustrated as mean ± SD, providing a clear representation of variability. Statistical analysis was carried out using one‐way ANOVA, followed by Tukey’s post hoc test for multiple comparisons.

Abbreviations: APTT, activated partial thromboplastin time; LPS, lipopolysaccharide; NOB, nobiletin; PAI‐1, plasminogen activator inhibitor; PT, prothrombin time.

^a^ *p* indicates a significant difference compared to the Control group.

^b^ *p* denotes significant differences compared to the NOB group.

^c^Denotes significant difference compared to the LPS group.

### 3.4. Effect of NOB in Mitigation of the Inflammatory Cytokine in LPS‐Induced ALI

The impact of NOB in reducing inflammation highlights its potential in mitigating the cytokine level. A single intranasal injection of LPS markedly elevated inflammatory cytokine levels of TNF‐α, IL‐6, GM‐CSF, and IL‐17 A in both plasma (Figure 3A–D) and lung tissue (Figure 3E–H) within 3 days when compared with the Control and NOB groups.

**Figure 3:**
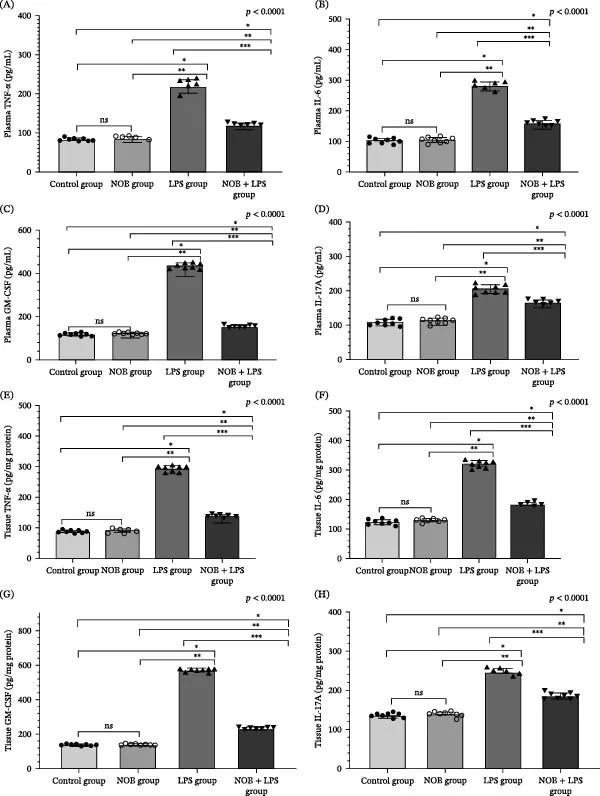
Effects of NOB on the inflammatory markers. (A) Plasma TNF‐alpha. (B) plasma IL‐6. (C) plasma GM‐CSF. (D) Plasma IL‐17A. (E) Tissue TNF‐alpha. (F) Tissue IL‐6. (G) Tissue GM‐CSF and (H) Tissue IL‐17A. The data are illustrated as mean ± SD, providing a clear representation of variability. Statistical analysis was carried out using one‐way ANOVA, followed by Tukey’s post hoc test for multiple comparisons. ns denotes nonsignificant,  ^∗^ *p* indicates a significant difference compared to the Control group, while  ^∗∗^ *p* denotes significant differences compared to the NOB group, and  ^∗∗∗^ denotes significant difference compared to the LPS group. GM‐CSF, granulocyte monocyte‐colony stimulating factor; IL‐17A, interleukin‐17A; IL‐6, interleukin‐6; LPS, lipopolysaccharide; NOB, nobiletin; TNF‐α, tumor necrosis factor‐alpha.

Pretreatment with NOB at a dose of 20 mg/kg effectively ameliorated the inflammatory responses. NOB significantly reduced the elevated cytokine levels in both plasma and lung tissue, demonstrating its anti‐inflammatory potential.

### 3.5. Effects of NOB in Mitigation Histopathological Alterations

H&E staining revealed preserved lung architecture in the Control and NOB groups, characterized by thin interalveolar septa and normal alveolar sacs. The septa contained numerous capillaries with minimal connective tissue.

Under the microscope, type‐I pneumocytes with flattened nuclei were distinctly visible, while type‐II pneumocytes appeared in limited numbers, identified by their centrally located vesicular nuclei lining the alveolar walls. The bronchioles were structured with a simple cuboidal epithelium, forming a wavy mucosal lining, supported by a well‐defined lamina propria (Figure 4A,B). Diffuse alveolar injury, vascular congestion, hyaline membrane formation, intra‐alveolar hemorrhage, fibrinoid deposits, inflammatory cell infiltration, edema, and early interstitial thickening suggestive of early fibrotic remodeling were observed in the LPS group.

**Figure 4:**
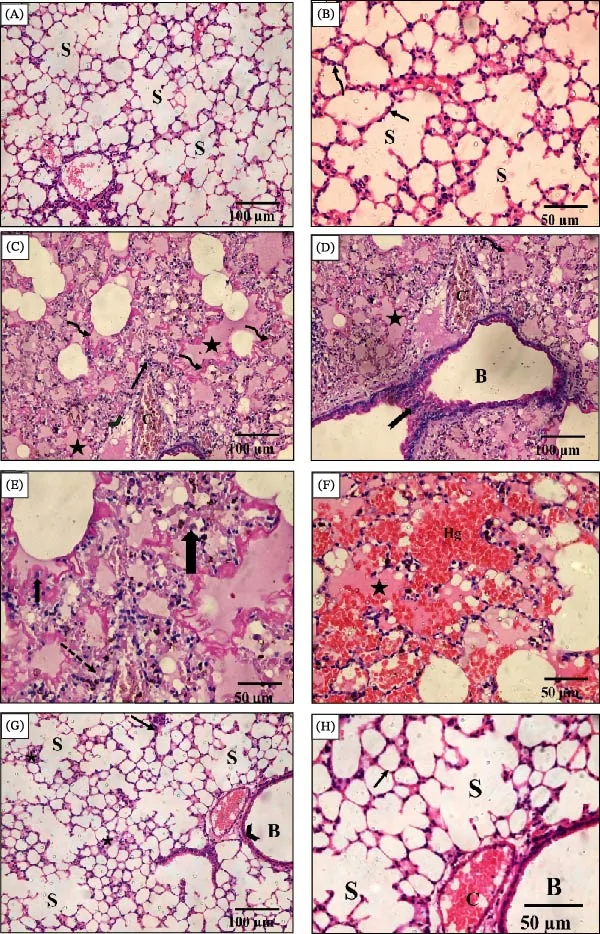
Light microscopic H&E‐stained photomicrographs of lung sections from the studied groups. (A, B) Control and NOB groups showing preserved normal lung architecture. The alveoli appeared as delicate, air‐filled sacs separated by thin interalveolar septa. The alveolar sacs (S) were surrounded by numerous capillaries and minimal connective tissue. The alveoli were predominantly lined by type I pneumocytes with flattened nuclei (black arrows), while scattered type II pneumocytes with rounded vesicular nuclei were also observed (curved arrows). (C–F) LPS group showing diffuse alveolar injury, vascular congestion (C), hyaline membrane formation (curved arrow), intra‐alveolar hemorrhage (Hg), fibrinoid deposits (arrowhead), inflammatory mononuclear cell infiltration (thin black arrow), edema (star), and early interstitial thickening suggestive of early fibrotic remodeling. Multinucleated giant cells (thick black arrow), reactive pneumocyte hyperplasia (dashed arrow), and bronchitis associated with submucosal congestion, epithelial denudation, and mononuclear cell infiltration (bifid arrow) were also observed. (G, H) NOB + LPS‐treated group showing marked attenuation of the histopathological alterations with partial preservation of the alveolar architecture, although mild interstitial hemorrhage and alveolar septal hypertrophy ( ^∗^) were still evident. H&E × 200, scale bar = 100 μm; H&E × 400, scale bar = 50 μm.

Bronchitis was noted together with submucosal congestion, epithelial denudation, and inflammation of mononuclear cells (Figure 4C–F). On the other hand, the NOB + LPS‐treated group demonstrated marked improvement in the histopathological appearance of the lung tissue, with preservation of the alveolar sacs and alveoli. Mild interstitial hemorrhage and alveolar septal hypertrophy were still evident (Figure 4G,H).

Additionally, to delineate the pulmonary iron accumulation at the cellular level, lung sections were subjected to staining with Perls’ Prussian blue. No discernible staining was observed in the tissues of the Control and NOB + LPS‐treated groups. Conversely, distinct staining patterns were noted in the LPS group, characterized by iron buildup in alveolar and interstitial immune cells (Figure 5A–D).

**Figure 5:**
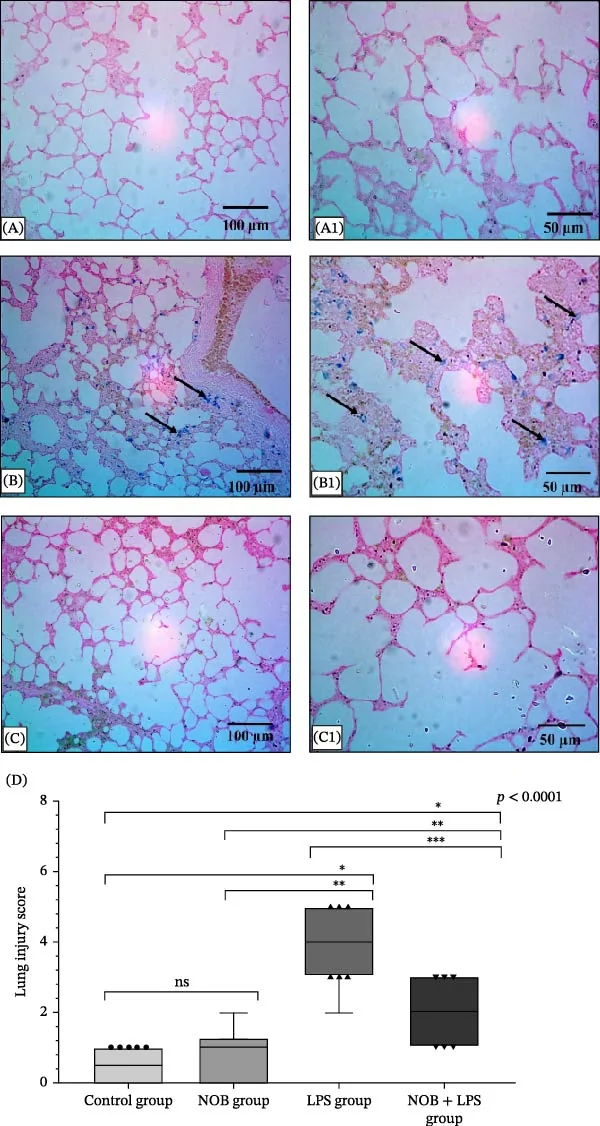
Light microscopic Perls’ Prussian blue‐stained photomicrographs of lung sections in all studied groups. (A, A1) Control and NOB groups showing negative staining in the tissues. (B, B1) LPS group showing distinct staining patterns characterized by significant iron buildup in alveolar and interstitial immune cells in the form of blue color (black arrows). (C, C1) NOB + LPS‐treated group showing negative staining in the tissues. Perls’ Prussian blue × 200, scale bar = 100 μm and Perls’ Prussian blue × 400, scale bar = 50 μm. (D) Effect of NOB on the lung injury scores induced by LPS among all studied groups. A boxplot was used to express the data. The bottom of the plot represents 25%, the middle represents the median, and the top represents 75% of the data. ns denotes nonsignificant,  ^∗^ *p* indicates a significant difference compared to the Control group, while  ^∗∗^ *p* denotes significant differences compared to the NOB group, and  ^∗∗∗^ denotes a significant difference compared to the LPS group, using Kruskal–Walli’s test followed by Dunn’s pairwise comparison post hoc test. LPS, lipopolysaccharide; NOB, nobiletin.

Histopathological analysis of lung sections from the NOB + LPS‐treated group demonstrated a marked reduction in inflammatory cell infiltration, with partial preservation of the bronchiolar, alveolar, and vascular architecture, together with a significant reduction in the lung injury score compared with the LPS group (Figure 5E).

### 3.6. Protective Effect of NOB Against LPS‐Induced Ultrastructural Alterations in Lung Tissue

The Control and NOB groups revealed a normal lung architecture under electron microscopy, maintaining their normal cellular integrity. Type I pneumocytes displayed a flattened, elongated shape, with the nuclei stretching across a large portion of the cytoplasm. In contrast, type II pneumocytes stood out with their cuboidal build, featuring euchromatic, spherical nuclei, and widely scattered microvilli. What truly set these cells apart was the abundance of lamellar bodies, essential for surfactant production and lung function.

Additionally, alveolar macrophages showcased large, indented nuclei accompanied by conspicuous nucleoli. Their cytoplasm was densely packed with lysosomes, while their surfaces were marked by numerous pseudopodia, highlighting their active immune role (Figure 6A,B).

**Figure 6:**
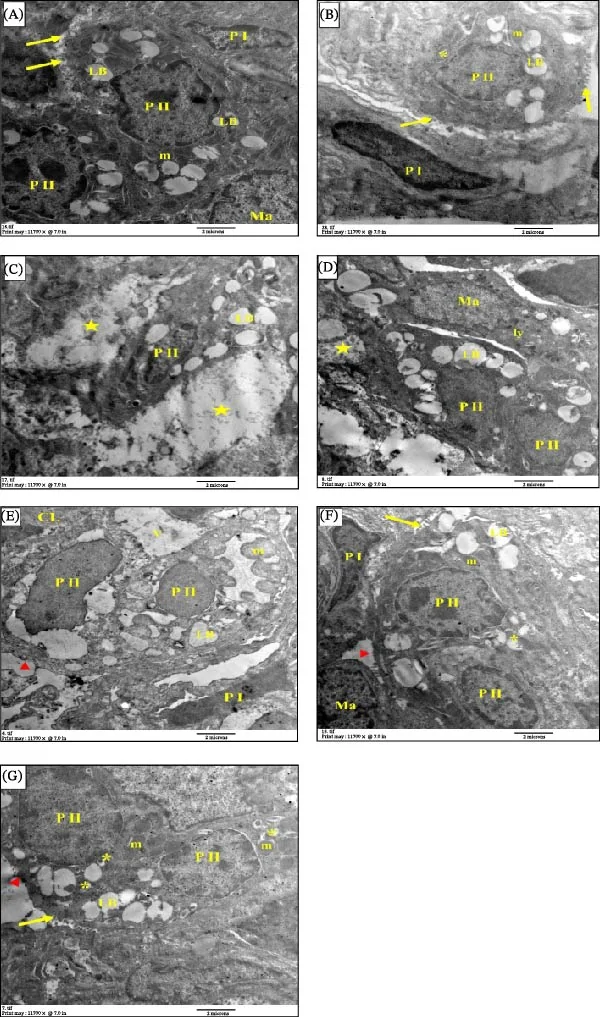
Representative transmission electron micrographs from lung tissue of the studied groups. (A, B) The Control and NOB groups exhibit type I pneumocytes (PI) characterized by elongated euchromatic nuclei encircled by a slender layer of cytoplasm. Type II pneumocytes (PII) exhibit rounded euchromatic nuclei and preserved microvillous boundaries (shown by arrows). Their cytoplasm has many mitochondria (m) and distinctive lamellar bodies (LB) with pronounced lamellae. (C–E) The LPS group exhibits type II pneumocytes (PII) characterized by irregularly shrunken nuclei, disrupted mitochondria (m), vacant lamellar bodies (LB), and disconnected microvilli (shown by the red arrowhead). Alveolar macrophages (AM) have distinctive indented nuclei, many lysosomes (Ly), and pseudopodia (MA). Collagen fibrils (CL) and edematous fluid (star) are deposited in the interstitial tissues. Type II pneumocytes (PII) have vacant lamellar bodies (LB) and a reduction in microvilli. (F) The NOB + LPS‐treated group has typical type II pneumocytes (PII) characterized by distinct cytoplasmic lamellar bodies (LB), mitochondria (m), and preserved microvilli (yellow arrow). (G) The NOB + LPS‐treated group exhibits type II pneumocytes (PII) characterized by abundant lamellar bodies (LB) containing surfactant ^∗^, intact microvilli (yellow arrow) in certain regions, numerous mitochondria (m), and large euchromatic nuclei with discernible nucleoli. Type I pneumocytes (PI) are observed. Nevertheless, there are limited regions exhibiting detached microvilli (shown by the red arrowhead). Magnification × 2000, scale bar = 2 μm.

In stark contrast, ultrathin sections from the LPS group painted a drastically altered landscape. Type II pneumocytes appeared distorted, with irregular, shrunken nuclei and hollow lamellar bodies, indicating a severe loss of function. Mitochondria were visibly damaged, while microvilli were completely detached, dispersing into the alveolar space. Adding to the destruction, the interstitial tissue was laden with thick collagen fibrils and edematous fluid, signifying profound tissue remodeling and inflammation (Figure 6C–E). The simultaneous administration of NOB and LPS enhanced lung ultrastructure, leading to type‐II pneumocytes characterized by enlarged euchromatic nuclei. A limited number of mitochondria remained compromised, but the microvilli were predominantly preserved. Some lamellar bodies were partially devoid of content, whereas others had strands of lung surfactant (Figure 6F,G).

### 3.7. Effect of NOB on Markers of Oxidative Stress in Lung Tissue During LPS‐Induced ALI

Three days following a single intranasal injection of LPS, there was infiltration and migration of inflammatory cells, which was confirmed by analyzing the MPO activity in lung samples. Figure 7A illustrates how LPS stimulation significantly raised MPO activity compared with the Control and NOB groups. In contrast to the LPS group, MPO activity was significantly reduced by pretreatment with NOB.

**Figure 7:**
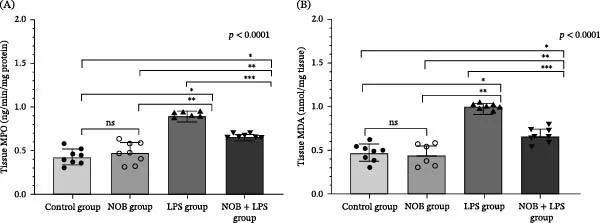
Effects of NOB on the activity of MPO and the production of MDA. (A) Tissue MPO, and (B) tissue MDA. Data are presented as mean ± SD. One‐way ANOVA test was performed, followed by Tukey’s post hoc test for multiple comparisons for the statistical comparison. ns denotes nonsignificant,  ^∗^ indicates a significant difference compared to the Control group, while  ^∗∗^ *p* denotes significant differences compared to the NOB group, and  ^∗∗∗^ denotes significant difference compared to the LPS group. LPS, lipopolysaccharide; MDA, malondialdehyde; MPO, myeloperoxidase; NOB, nobiletin.

Additionally, oxidative indicators such as MDA (lipid peroxidation marker) were assessed to evaluate the antioxidative properties of NOB. Figure 7B illustrates that LPS significantly increased the MDA content of lung tissue compared with the Control and NOB groups. In contrast, administration of NOB notably exhibited a significant decrement of MDA content in lung tissue in the NOB + LPS group compared to the LPS group. On the other hand, NOB administration significantly attenuated the LPS‐induced reduction in SOD activity in the treated group compared with the LPS group, as shown in Table 4.

**Table 4:** Effects of NOB on the lung tissue levels of SP‐A, angiotensin II, angiotensin 1–7, ACE2 activity, SOD, and complex I activities.

Parameters/groups	Control group (*n* = 8)	NOB group (*n* = 8)	LPS group (*n* = 8)	NOB + LBS group (*n* = 8)	*F* ratio	*p*‐Value
SP‐A ng/mg protein	0.62 ± 0.08	0.63 ± 0.09	0.34 ± 0.016^ab^	0.45 ± 0.02^abc^	36.26	*p* < 0.0001
Angiotensin II pg/mg protein	78.38 ± 6.74	75.38 ± 8.48	259.13 ± 38.81^ab^	164.34 ± 10.46^abc^	139.65	*p* < 0.0001
Angiotensin 1–7 pg/mg protein	383.63 ± 26.81	401.75 ± 40.10	131.75 ± 15.43^ab^	235.13 ± 36.20^abc^	135.69	*p* < 0.0001
ACE2 activity ng/min/mg protein	133.00 ± 8.49	142.13 ± 7.49	55.25 ± 11.11^ab^	85.00 ± 5.50^abc^	190.49	*p* < 0.0001
Complex I activity (nmol/min/mg protein)	52.91 ± 1.9	51.78 ± 2.4	32.34 ± 1.34^ab^	52.14 ± 1.02^c^	36.95	*p* < 0.0001
SOD (activity units/mg protein)	1.12 ± 0.16	1.3 ± 0.24	0.31 ± 0.17^ab^	1.01 ± 0.32^c^	42.91	*p* < 0.0001

*Note:* Data are presented as mean ± SD. One‐way ANOVA test was performed, followed by Tukey’s post hoc test for multiple comparisons for the statistical comparison.

Abbreviations: ACE2, angiotensin converting enzyme 2; LPS, lipopolysaccharide; NOB, nobiletin; SOD, superoxide dismutase; SP‐A, surfactant protein‐A.

^a^Indicates a significant difference compared to the Control group.

^b^ *p* denotes significant differences compared to the NOB group.

^c^Denotes significant difference compared to the LPS group.

### 3.8. Effect of NOB on SP‐A, Angiotensin II and Angiotensin 1–7 Levels, ACE2 Activity, and Complex I Activity in Lung Tissue in Lung Tissue During LPS Induced ALI

Table 4 shows that, 3 days following a single intranasal injection of LPS, there was a significant decrease in pulmonary SP‐A content and ACE2 activity with a concomitant significant increase in pulmonary angiotensin II and angiotensin 1–7 levels when compared with the Control and NOB groups. In contrast to the LPS group, pulmonary SP‐A content and ACE2 activity were significantly elevated with concomitant significant decrease in pulmonary angiotensin II and angiotensin 1–7 levels by pretreatment with NOB at a dose of 20 mg/kg for 1 week prior to LPS administration. The treatment also upregulated the level of mitochondrial complex I, as shown in Table 4.

### 3.9. Effect of NOB on Modulating LPS‐Induced Inflammatory Pathway miR‐200, AT1R/p38 MAPK

To explore the protective mechanisms of NOB, qPCR analysis was conducted on key inflammatory pathways that mediate cytokine production and immune cell recruitment. miR‐200, a critical regulator of the inflammatory cascade, interacts with ATR1. As shown in Figure 8A,B, a single intranasal injection of LPS (15 mg/kg) obviously upregulates the relative mRNA expression of miR‐200 and ATR1. Furthermore, in Figure 8C the levels of protein expression for p38 MAPK, the downstream signaling pathway of ATR1, were also increased following LPS administration, as compared to the Control and NOB groups.

**Figure 8:**
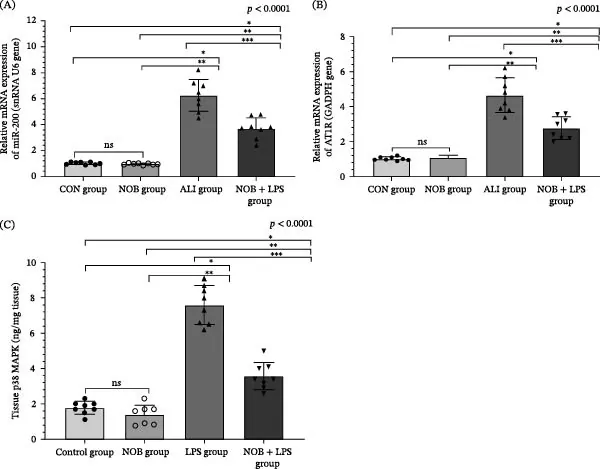
Effects of NOB on relative mRNA expression of miR‐200 and AT1R with tissue protein level of p38 MAPK. (A) Relative mRNA expression of miR‐200. (B) relative mRNA expression of AT1R. (C) tissue MAPK. Data are presented as mean ± SD. One‐way ANOVA test was performed, followed by Tukey’s post hoc test for multiple comparisons for the statistical comparison.ns denotes nonsignificant,  ^∗^ *p* indicates a significant difference compared to the Control group, while  ^∗∗^ *p* denotes significant differences compared to the NOB group, and  ^∗∗∗^ denotes a significant difference compared to the LPS group. AT1R, angiotensin II receptor type 1; GADPH, glyceraldehyde‐3‐phosphate dehydrogenase; LPS, lipopolysaccharide; miR‐200, microRNA‐200; NOB, nobiletin; p38 MAPK, p38 mitogen‐activated protein kinase.

However, the anti‐inflammatory and protective effects of NOB were highlighted at a dose of 20 mg/kg for 1 week prior to LPS administration as it significantly attenuated the expression of miR‐200, which was accompanied by reduced AT1R expression and lower tissue p38 MAPK levels. This was evident from the significant decrement of ATR1 relative mRNA expression. As a result, the downstream inflammatory signaling pathway, including p38 MAPK, was markedly decreased in the NOB + LPS group as compared to that in the LPS group.

### 3.10. Effect of NOB on LPS‐Induced Exaggeration of HMGB1/NF‐κB/mTOR Pathways

To further explore the potential mechanistic pathways, the relative mRNA expression of the HMGB1‐mediated/mTOR pathway was examined, together with assessment of the immunohistochemical expression of NF‐κB in lung tissue. The relative mRNA expression of HMGB1 together with tissue levels of mTOR were obviously increased by a single intranasal injection of LPS (15 mg/kg) when compared with the Control and NOB groups, as illustrated in Figure 9A,B. This was accompanied by intense positive immunohistochemical staining of NF‐κB in the LPS group compared to negative expression of NF‐κB in the Control and NOB groups, as shown in Figure 10A–D.

**Figure 9:**
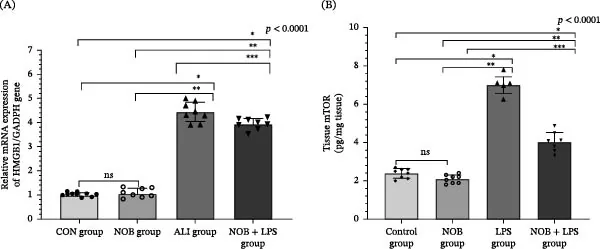
Effects of NOB on relative mRNA expression of HMGB1/mTOR pathways. (A) Relative mRNA expression of HMGB1/GADPH. (B) tissue mTOR. Data are presented as mean ± SD. One‐way ANOVA test was performed, followed by Tukey’s post hoc test for multiple comparisons for the statistical comparison. ns denotes nonsignificant,  ^∗^ *p* indicates a significant difference compared to the Control group, while  ^∗∗^ *p* denotes significant differences compared to the NOB group, and  ^∗∗∗^ *p* denotes a significant difference compared to the LPS group. GADPH, glyceraldehyde‐3‐phosphate dehydrogenase; HMGB1, high mobility group box 1 protein; LPS, lipopolysaccharide; mTOR, mammalian target of rapamycin; NOB, nobiletin.

**Figure 10:**
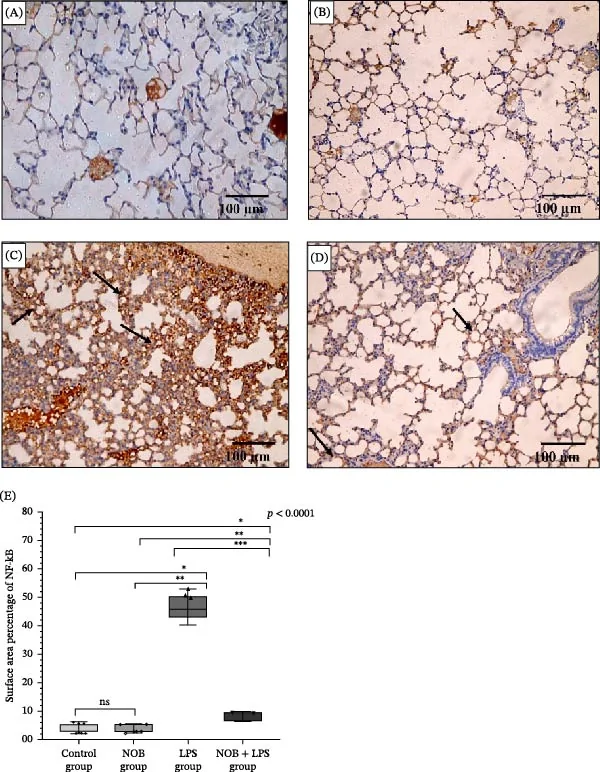
Light microscopic photomicrographs of NF‐кB immunohistochemical‐stained lung tissue in all studied groups. (A, B) Control and NOB groups showing negative expression of NF‐кB immune reaction in all cells. (C) LPS group shows a strong positive immunoreactivity (yellow arrows). (D) NOB + LPS‐treated group showing weak immune reaction in most cells (arrows). NF‐кB immune reaction in cells immunostaining ×200, scale bar = 100 µm. (E) Surface area % of NF‐кB among all studied groups. A boxplot was used to express the data. The bottom of the plot represents 25%, the middle represents the median, and the top represents 75% of the data. ns denotes nonsignificant,  ^∗^ *p* indicates a significant difference compared to the Control group, while  ^∗∗^ *p* denotes significant differences compared to the NOB group, and  ^∗∗∗^ denotes a significant difference compared to the LPS group using the Kruskal–Wallis test followed by Dunn’s pairwise comparison post hoc test. LPS, lipopolysaccharide; NOB, nobiletin.

However, pretreatment with NOB exerts a notable anti‐inflammatory effect through dramatic downregulation of HMGB1 relative mRNA expression and suppression of mTOR levels when compared with the LPS group. This is in accordance with marked reduction in the immunohistochemical staining of NF‐κB in the NOB + LPS group as compared to the LPS group (Figure 10E).

## 4. Discussion

ALI is a critical, life‐threatening lung condition characterized by intense pulmonary inflammation, increased vascular permeability, and impaired alveolar function [24]. This research set out to explore the protective potential of NOB, a natural flavonoid, in mitigating LPS‐induced ALI in a rat model.

The LPS‐induced model reproduces many of the inflammatory and pathological features of experimental ALI through activation of multiple signaling pathways, leading to excessive cytokine production, oxidative stress, tissue injury, and coagulopathy [25]. Consistent with this model, LPS administration markedly increased the circulating and pulmonary levels of TNF‐α, IL‐6, GM‐CSF, and IL‐17A, confirming the establishment of a robust inflammatory response.

The relatively high intranasal LPS dose (15 mg/kg) used in the present study was intentionally selected to establish a reproducible model of severe experimental ALI characterized by marked inflammatory and histopathological alterations, as reported in previous studies [1]. Although this model reproduces many key inflammatory and histopathological features of ALI, it represents an acute experimental condition and does not fully recapitulate the heterogeneity or clinical spectrum of human ALI/acute respiratory distress syndrome (ARDS) [4, 25].

LPS‐induced ALI involves the activation of TLR4 through its interaction with LPS‐binding protein, myeloid differentiation factor 2 (MD‐2), and cluster differentiation factor 14 (CD14). This trigger signaling cascades via myeloid differentiating factor 88 (MyD88), leading to NF‐κB activation [26]. HMGB1 amplifies the TLR4/NF‐κB pathway, activating p38 MAPK and promoting inhibitor of nuclear factor kappa B (IκB‐α) degradation, enabling NF‐κB to translocate to the nucleus. This process triggers the release of proinflammatory cytokines such as TNF‐α, IL‐6, IL‐17, and GM‐CSF, exacerbating inflammation and lung injury [27].

In addition, LPS‐induced upregulation of AT1R activates the p38 MAPK/JAK/STAT signaling cascade, further promoting inflammatory cytokine transcription and amplifying the inflammatory response [28]. These cytokines, in turn, sustain AT1R activation, creating a positive feedback loop that perpetuates inflammation, oxidative stress, and vascular remodeling [29]. In agreement with these mechanisms, the present study demonstrated characteristic histopathological changes, including diffuse alveolar injury, inflammatory cell infiltration, alveolar hemorrhage, and increased MPO activity, reflecting enhanced neutrophil infiltration within the lung tissue [14].

Consistent with these inflammatory mechanisms, the present study demonstrated significant upregulation of miR‐200, AT1R, HMGB1, p38 MAPK, mTOR, and NF‐κB following LPS administration. NOB treatment markedly attenuated these molecular alterations, suggesting modulation of multiple interconnected inflammatory signaling pathways involved in ALI pathogenesis.

NOB treatment was associated with reduced AT1R expression and lower tissue p38 MAPK levels, suggesting that modulation of the AT1R/p38 MAPK axis may contribute to its protective effects against LPS‐induced lung injury. These effects highlight NOB’s ability to disrupt key proinflammatory signaling cascades, providing significant protection against lung injury [1].

Targeting and disruption of the inflammatory pathways by NOB was confirmed in the present study by qRT‐PCR analysis that demonstrated significant downregulation of AT1R, in addition to lowering of tissue p38 MAPK. NOB treatment was associated with reduced HMGB1 expression and decreased NF‐κB immunostaining. These findings are consistent with attenuation of inflammatory signaling, although direct effects of NOB on the HMGB1–TLR4 interaction or NF‐κB activation were not directly assessed. Through this dual mechanism, it suppresses the production of proinflammatory cytokines.

To gain deeper insight into how NOB mitigates LPS‐induced ALI, we investigated its effects on mTOR. In LPS‐induced ALI, HMGB1 promotes the activation of PI3K/Akt, leading to the phosphorylation of downstream targets such as mTOR [30]. This pathway plays a crucial role in cell survival when exposed to oxidative stress and plays a dual role: while its activation prevents epithelial cell apoptosis and supports cell survival, overactivation can exacerbate inflammatory responses and tissue remodeling through suppression of autophagy and subsequent excessive ROS formation that acts as a signaling molecules that activate proinflammatory pathways, such as NF‐κB [8]. Results of the current study: NOB significantly reduced mTOR. NOB treatment was associated with reduced HMGB1 expression and lower tissue mTOR levels. Given the established relationship between HMGB1‐related signaling and the PI3K/Akt/mTOR pathway, these findings may be consistent with modulation of this pathway; however, the intermediate signaling events were not directly assessed in the present study [31].

Based on the previous mechanisms, NOB effectively attenuated proinflammatory cytokines, including TNF‐α, IL‐6, GM‐CSF, and IL‐17A, measured in plasma and lung tissue homogenates. This suppression was further supported by diminished NF‐κB immunostaining, consistent with reduced tissue expression of NF‐κB, indicating attenuation of NF‐κB activation in lung tissue, together with reduced MPO activity and histopathological inflammatory cell infiltration [14].

The ACE2/angiotensin 1–7 axis plays a crucial role in mitigating endothelial dysfunction [32]. To assess its involvement, levels of angiotensin II, angiotensin 1–7, and ACE2 activity in lung tissue were measured, along with qRT‐PCR analysis of miR‐200. Additionally, coagulopathy, a common complication of ALI, is exacerbated by hyperinflammation, platelet hyperactivation due to endothelial dysfunction, and impaired fibrinolysis. The cytokine storm, decreased ACE2, and increased thrombotic factors contribute to excessive clot formation, thereby elevating the risk of thrombosis and microvascular complications. To evaluate coagulopathy, plasma markers, including D‐Dimer, PAI‐1, PT, APTT, and ferritin, were measured [33].

The observed increase in miR‐200 expression was accompanied by alterations in ACE2/Ang axis markers, suggesting a possible interaction between inflammatory signaling and RAS dysregulation [5].

NOB treatment attenuated the LPS‐induced alterations in miR‐200 expression, ACE2 activity, Ang 1–7, and Ang II levels. These changes may contribute to reduced AT1R‐mediated oxidative stress and improved pulmonary vascular integrity. The effects of NOB on vascular stability were further evidenced by reductions in markers of coagulopathy, including D‐Dimer, PAI‐1, PT, APTT, and ferritin. This reduction in coagulopathy was consistent with decreased alveolar hemorrhage noted in histological evaluations, together with reduction of iron buildup in alveolar and interstitial immune cells, as indicated by Perls’ Prussian blue‐stained photomicrographs. This emphasizes its protective effects.

To further evaluate the impact of NOB on surfactant homeostasis, SP‐A levels were measured in the lung tissue. “SP‐A levels were significantly reduced in LPS‐treated animals” [1]. NOB treatment attenuated the LPS‐induced reduction in SP‐A levels, as demonstrated by the ELISA. The increased SP‐A levels in the NOB + LPS group may indicate better preservation of surfactant homeostasis and attenuation of LPS‐induced lung injury.

To assess oxidative stress and mitochondrial function, we measured MDA levels, SOD, and complex I activities in lung tissue, respectively. LPS administration resulted in a significant increase in MDA levels combined with the attenuation of SOD and complex I activities. However, the NOB ability to inhibit AT1R‐mediated ROS was reflected by significant decrement of lipid peroxidation, neutrophil infiltration, and partial preservation of SOD and complex I activities.

Histological and ultrastructural analyses further supported the protective effects of NOB. Lung tissues from the NOB + LPS‐treated group showed marked attenuation of the histopathological alterations, with reduced inflammatory cell infiltration and alveolar hemorrhage, together with partial preservation of the alveolar architecture compared with the extensive damage observed in the LPS‐treated group. TEM further supported these findings by demonstrating improved preservation of the cellular ultrastructure.

Pulmonary edema, a hallmark characteristic of ALI, was evaluated by measuring the lung wet‐to‐dry weight ratio, which showed a significant increase in LPS‐treated animals. As a result of the previous mechanisms, NOB treatment significantly reduced the lung wet‐to‐dry ratio, indicating the attenuation of pulmonary edema. This effect was accompanied by reduced inflammatory and coagulation‐related markers, although the specific mechanism underlying the reduction in pulmonary edema was not directly established.

The mortality observed in the LPS‐treated group likely reflects the severity of the experimental ALI model induced by the selected LPS dose, as similar mortality rates have been reported in previous studies using comparable intranasal LPS protocols [1]. Although NOB treatment was associated with improved survival, the relatively small sample size precludes definitive conclusions regarding its survival benefit.

Collectively, these findings suggest that NOB exerts protective effects against LPS‐induced ALI through the modulation of inflammatory, oxidative, and vascular injury‐associated markers.

However, the present study has several limitations that should be acknowledged. First, although NF‐κB signaling appeared to be a central component of the proposed mechanism, additional experimental validation should be performed. Second, the mechanistic interactions of NOB with inflammatory signaling pathways were inferred from molecular, biochemical, and histopathological findings without the use of pathway‐specific pharmacological inhibitors or genetic approaches, which would provide stronger evidence. Third, although the LPS‐induced ALI model reproduces many of the inflammatory and pathological features of experimental ALI, it does not fully recapitulate the complexity of human ALI or ARDS. Furthermore, downstream targets of miR‐200, such as ZEB1/ZEB2 and KEAP1, were not evaluated, and fibrosis‐specific molecular markers or collagen‐specific staining were not included to further characterize the early tissue remodeling observed in this model.

Future studies should further investigate the molecular mechanisms underlying the protective effects of NOB, including its downstream targets and signaling interactions, evaluate its potential synergistic effects with established therapeutic approaches, determine its optimal dosing strategy and long‐term safety in preclinical models, and subsequently assess its pharmacokinetic profile and therapeutic efficacy in well‐designed clinical trials for ALI.

## 5. Conclusion

The anti‐inflammatory and vascular‐stabilizing properties of NOB suggest its potential utility in mitigating ALI through the potential involvement of molecular inflammatory pathways, including AT1R/p38 MAPK and HMGB1/NF‐κB/mTOR. Additionally, the restoration of ACE2 activity and Ang 1–7 levels may be associated with vascular stability, thereby reducing endothelial dysfunction and coagulopathy.

Based on these findings, NOB represents a promising candidate for further preclinical investigation as a potential therapeutic strategy for ALI owing to its combined anti‐inflammatory, antioxidant, endothelial protective, and vascular stabilizing properties.

## Author Contributions

Rehab Ahmed Ahmed El‐Shaer, Sarah Ibrahim, and Marwa Mahmoud Awad conceived and designed the research and prepared figures. Rehab Ahmed Ahmed El‐Shaer, Sarah Ibrahim, Norhan Ahmed AbuoHashish, Passant Medhat Hewady, Rowida Raafat Ibrahim, Omnia Safwat El‐Deeb, Amira K. Eltokhy, Eman E. Farghal, Radwa Eissa, Marwa M. Shalaby, Heba Bassiony Ghanem, and Marwa Mahmoud Awad performed experiments and interpreted the results of experiments. Rehab Ahmed Ahmed El‐Shaer, Sarah Ibrahim, Rowida Raafat Ibrahim, Omnia Safwat El‐Deeb, Norhan Ahmed AbuoHashish, and Marwa Mahmoud Awad analyzed the data. Rehab Ahmed Ahmed El‐Shaer, Sarah Ibrahim, Norhan Ahmed AbuoHashish, Passant Medhat Hewady and Marwa Mahmoud Awad drafted the manuscript. Rehab Ahmed Ahmed El‐Shaer, and Marwa Mahmoud Awad edited and revised the manuscript.

## Funding

The authors did not receive any specific funding from financial sources for this research.

## Disclosure

The authors approved the final version of the manuscript.

## Conflicts of Interest

The authors declare no conflicts of interest.

## Data Availability

The corresponding author will provide the data supporting the study’s findings upon request.
